# Correlation Network Analysis Applied to Complex Biofilm Communities

**DOI:** 10.1371/journal.pone.0028438

**Published:** 2011-12-07

**Authors:** Ana E. Duran-Pinedo, Bruce Paster, Ricardo Teles, Jorge Frias-Lopez

**Affiliations:** 1 Forsyth Institute, Cambridge, Massachusetts, United States of America; 2 Harvard School of Dental Medicine, Boston, Massachusetts, United States of America; Argonne National Laboratory, United States of America

## Abstract

The complexity of the human microbiome makes it difficult to reveal organizational principles of the community and even more challenging to generate testable hypotheses. It has been suggested that in the gut microbiome species such as *Bacteroides thetaiotaomicron* are keystone in maintaining the stability and functional adaptability of the microbial community. In this study, we investigate the interspecies associations in a complex microbial biofilm applying systems biology principles. Using correlation network analysis we identified bacterial modules that represent important microbial associations within the oral community. We used dental plaque as a model community because of its high diversity and the well known species-species interactions that are common in the oral biofilm. We analyzed samples from healthy individuals as well as from patients with periodontitis, a polymicrobial disease. Using results obtained by checkerboard hybridization on cultivable bacteria we identified modules that correlated well with microbial complexes previously described. Furthermore, we extended our analysis using the Human Oral Microbe Identification Microarray (HOMIM), which includes a large number of bacterial species, among them uncultivated organisms present in the mouth. Two distinct microbial communities appeared in healthy individuals while there was one major type in disease. Bacterial modules in all communities did not overlap, indicating that bacteria were able to effectively re-associate with new partners depending on the environmental conditions. We then identified hubs that could act as keystone species in the bacterial modules. Based on those results we then cultured a not-yet-cultivated microorganism, *Tannerella sp.* OT286 (clone BU063). After two rounds of enrichment by a selected helper (*Prevotella oris* OT311) we obtained colonies of *Tannerella* sp. OT286 growing on blood agar plates. This system-level approach would open the possibility of manipulating microbial communities in a targeted fashion as well as associating certain bacterial modules to clinical traits (e.g.: obesity, Crohn's disease, periodontal disease, etc).

## Introduction

Knowledge of qualitative and quantitative data of complex microbial communities is necessary to initially characterize system processes in the environment. These processes are determined by many functionally diverse, differently active sets of the microbial species that form the community. In turn, the microbial community responds to changes by modifying its composition or adapting their gene expression profiles to the new environment. Accumulation of array and metagenomic data has shed light on the composition of microbial communities from different environments [1]–[5]. However, little it is known about their organization and the principles that govern the associations of the different species.

Systems biology techniques have been applied to explain the functional organization of a variety of biological systems, bridging the gap from individual elements to systems biology by exploring the observed relationships between the individual elements of the system. Among these techniques, network analysis models have been widely used. A classical application has been the study of cellular systems interactions among and between cellular elements (e.g. proteins) of a biological system [6]–[8]. Different tools for network analysis have been developed depending on the topic of interest. Furthermore, using network analysis it is possible to identify influential individuals within a group. For instance, a regulatory network centrality analysis will single out which element or elements regulate many others in the system and could be considered global regulators of the system.

One set of tools available is correlation network analysis, which are unique in the sense that they are not the result of direct experimental data but determined by collecting large amounts of data and calculating the correlation between all elements [6], [7]. These methods have been successfully applied to the study of various biological contexts including cancer [9], evolutionary relationships [10] and yeast genetics [11]. Recently Steele et al. have studied linkages within a microbial plankton community using co-occurrence patterns determined by either automated ribosomal intergenic spacer analysis (ARISA) or terminal restriction length polymorphism (TRFLP) [12].

In order to gain an understanding of the organization of a complex microbial community, we used correlation network analysis to study the organization and bacterial interactions in the oral plaque, in health and disease. Recently, Zhou et al. using Pearson's correlation matrix reconstructed a molecular ecological network in soil microbial communities [13] and Gilbert et al. used correlation network analysis to study microbial community dynamics in the marine environment [14]. To our knowledge weighted correlation network analysis has not been previously used to the study of bacterial associations in microbial communities. Beyond its basic research interest, we show that the use of network analysis on microbial communities has practical applications. Studying the centralities of the network we may identify potential target organisms (keystone species) whose disappearance might lead to the disturbance of a mature biofilm. Moreover, we showed that network analysis facilitated the cultivation of a previously uncultivated organism by analyzing key relationships among uncultivated organisms.

## Results

In order to characterize the microbial communities we used results from two different methodologies: one was the checkerboard DNA-DNA hybridization technique [15] that identifies only important cultivable oral bacteria and the other the Human Oral Microbe Identification Microarray (HOMIM) [3]. Checkerboard hybridization detected 40 cultivable periodontal species while HOMIM detected a total of 274 species or clusters of species of oral bacteria including not-yet-cultivated species. Checkerboard data was obtained from 2,565 individual subgingival plaque samples from patients with periodontitis while for the HOMIM analysis results came from 90 sites from healthy individuals and 514 sites from individuals with periodontitis. The raw and normalized intensities were made publicly available by submission to GEO [16] and can be accessed via accession number GSE32159.

The first step of the analysis was estimating the missing values in our data set. Most microarray based technologies suffer from frequent missing values due to various experimental reasons. Since the missing data points can hinder downstream analyses a wide variety of techniques have been developed to deal with missing values in large-scale data sets. It is not reasonable to simply discard such observations or remove the corresponding cases, since this will lose valuable information and can lead to selection bias; instead, the missing values need to be replaced or predicted as accurately as possible before the actual data analysis. We estimated the missing values using a bayesian principal component analysis (BPCA) method that has been shown to perform better than other methods estimating missing values in microarrays [17]. The estimated results were used for the next series of analysis.

### Correlation network analysis of bacterial communities using Weighted Correlation Network Analysis (WGCNA)

To first test the biological meaningfulness of the modules obtained by WGCNA analysis we used a checkerboard DNA-DNA hybridization database because associations among the species contained in the array have been widely studied in the past [15], [18]–[20].

For checkerboard analysis the power of the pairwise Pearson correlation was β = 9 with scale free topology R^2^ = 0.4 (the maximum for these samples). The low R^2^ value is probably due to the low number of species in the dataset. Hierarchical clustering led to the removal of 9 outlier samples and a total of 2,556 checkerboard arrays samples used. Interestingly, we identified a single cluster, which represented a unique microbial community associated with disease (Figure S1a).

**Figure 1:**
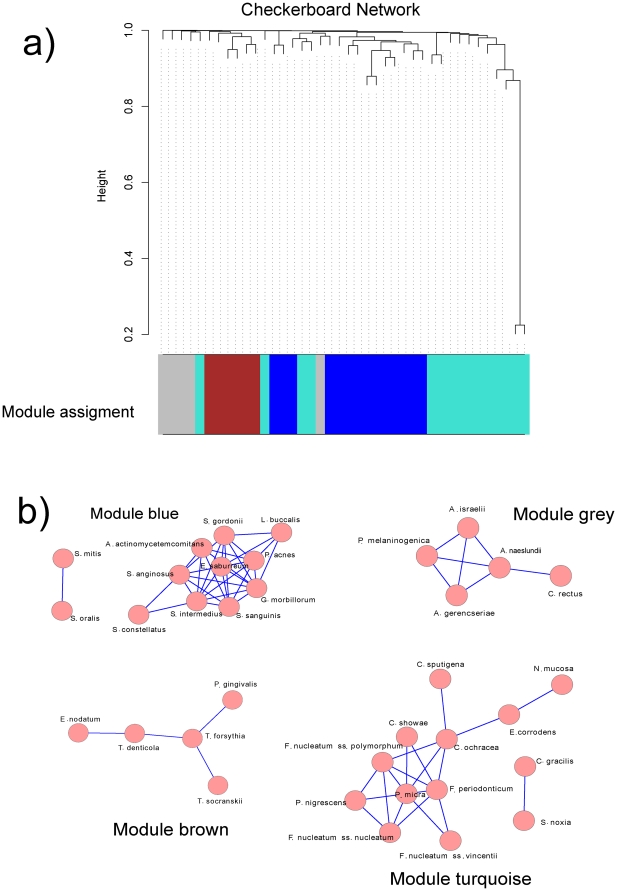
WGCNA correlation network results of bacterial species in checkerboard hybridization results. The images show the Cytoscape representation of the correlation networks for the 4 modules identified by WGCNA. Checkerboard analysis was performed for 40 species of oral bacteria on a total of 2,565 individual tooth from patients with periodontitis. R^2^ used for scale free topology model fit was 0.40, the maximum value in the analysis. The identified modules correlated well with microbial complexes previously described [20].

Using WGCNA we identified 4 bacterial modules that arbitrarily were given the colors blue (12 species), brown (5 species), grey (5 species) and turquoise (13 species) (Fig. 1).

We then expanded our analysis using results from the HOMIM, with samples from healthy and diseased individuals [3]. HOMIM results from healthy individuals in cluster 1 (51 samples) had a power of the pairwise Pearson correlation β = 5 with scale free topology R^2^ = 0.9. HOMIM results from healthy individuals in cluster 2 (37 samples) had β = 6 with scale free topology R^2^ = 0.85. Finally, HOMIM results from diseased individuals in cluster 1 (467 samples) had β = 7 with scale free topology R^2^ = 0.9 and HOMIM results from diseased individuals in cluster2 (47 samples) had β = 7 with scale free topology R^2^ = 0.85. We obtained high values of R^2^, although when the network is small (with few species) or the many species are highly correlated with each other scale-free fit may not be possible to achieve.

The HOMIM microarray includes species that have not been cultured yet and that could be important in the development of the oral biofilm and disease progression. Hierarchical clustering led to the removal of 2 outlier samples and the identification of two clusters of similar size in the samples from healthy individuals, which represented two different distinct microbial communities associated with health (Figure S1b). In the case of the samples from disease no outliers were detected and 2 clusters were identified. Nonetheless, contrary to what happened in the samples from healthy individuals, one cluster had 10 fold more samples than the other (467 vs. 47 samples) which implies that there is a singular bacterial community frequently associated with disease (Figure S1a and c). This community is more complex than any of the other community profiles obtained from the other clusters (Figure S2).

**Figure 2:**
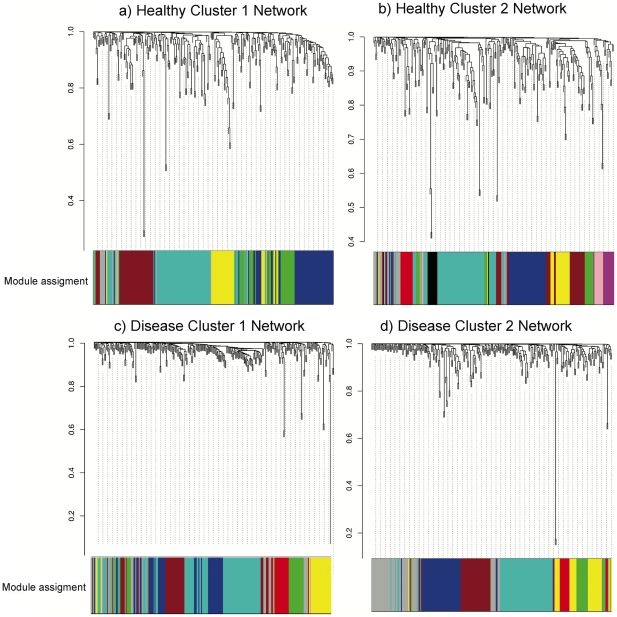
WGCNA correlation network results of bacterial species in healthy and diseased individuals from HOMIM results. Clustering dendrogram of species, with dissimilarity based on topological overlap, together with assigned module colors. a) Cluster 1 from healthy individuals (51 samples), R^2^ used for scale free topology model fit was 0.90 and a total of 6 bacterial modules were identified. b) Cluster 2 from healthy individuals (37 samples), R^2^ used for scale free topology model fit was 0.85 and a total of 10 bacterial modules were identified. c) Cluster 1 from diseased individuals (467 samples), R^2^ used for scale free topology model fit was 0.90 and a total of 6 bacterial modules were identified. D) Cluster 2 from diseased individuals (49 samples), R^2^ used for scale free topology model fit was 0.85 and a total of 7 bacterial modules were identified.

We then proceeded to identify the bacterial modules (groups of bacterial species that appeared associated across samples). Figure 2 summarizes the results of module identification in health (Fig. 2a and 2b) and disease (Fig. 2c and 2d). Additionally, we tried to obtain consensus networks using the combined results of healthy and diseased samples. However, the structure of the networks in health and disease was so different that it was not possible to obtain any consensus network. Even within groups (health and disease) it was not possible to obtain consensus networks.

### Network centralities and identification of hubs

Table 1 shows the overall statistics of centralities for the identified modules. Interestingly, modules in clusters from healthy biofilms present lower centralization and higher density than the modules in the clusters from diseased biofilms, which may indicate that those modules could be more resilient to changes and the correlations among their members are high.

**Table 1:** Fundamental statistics describing the networks.

Samples	Module	Clustering coefficient	Network centralization	Network density	Avg. number of neighbors	Number of nodes
**Checkerboard**	Blue	0.72	0.27	0.05	5.5	12
	Brown	0.0	0.58	0.4	1.6	5
	Grey	0.7	0.5	0.7	2.8	5
	Turquoise	0.47	0.37	0.27	3.23	13
**HOMIM Healthy Cluster 1**						
	Blue	0.911	0.153	0.852	52.8	63
	Brown	0.775	0.319	0.579	24.1	44
	Green	0.802	0.291	0.667	22.7	35
	Grey	0.751	0.182	0.346	4.2	13
	Turquoise	0.689	0.454	0.469	37.6	81
	Yellow	0.821	0.298	0.662	23.8	37
**HOMIM Healthy Cluster 2**						
	Black	0.806	0.269	0.659	9.7	15
	Blue	0.906	0.158	0.827	37.2	46
	Brown	0.873	0.202	0.746	23.1	32
	Green	0.860	0.279	0.693	11.8	18
	Grey	0.451	0.221	0.216	3.7	18
	Magenta	0.928	0.115	0.901	11.7	14
	Pink	0.913	0.192	0.835	10.9	14
	Red	0.859	0.324	0.717	10.8	16
	Turquoise	0.828	0.322	0.634	48.2	77
	Yellow	0.960	0.062	0.944	19.9	22
**HOMIM Disease Cluster 1**						
	Blue	0.837	0.353	0.593	48.1	82
	Green	0.736	0.373	0.495	12.4	26
	Grey*	0.291	0.324	0.183	2.7	16
	Red	0.768	0.400	0.464	8.8	21
	Turquoise	0.895	0.226	0.768	67.6	89
	Yellow	0.715	0.385	0.336	8.7	27
**HOMIM Disease Cluster 2**						
	Blue	0.705	0.423	0.398	15.9	41
	Brown	0.841	0.279	0.708	26.2	38
	Green	0.441	0.225	0.249	5.5	23
	Grey	0.453	0.243	0.175	4.4	26
	Red*	0.526	0.389	0.382	3.8	11
	Turquoise	0.483	0.294	0.139	11.0	81
	Yellow	0.787	0.374	0.538	14.0	27

These concepts describe the overall shape and centralities of the modules. The Clustering coefficient is a measure of local connections. Network centralization describes whether the network is dominated by a few central nodes or not. Network density assess the proportion of ties in a network relative to the total number possible. Finally, the average number of neighbors and number of nodes describe the size and interconnectedness of the module.

*Only subset of nodes connected.

Next step was to identify hubs in each of the modules. The question of which network elements are the most important cannot be answered unambiguously. Ranking nodes (species) in the network is accomplished by measuring different centrality indices using different algorithms. We used three different algorithms. First, we used degree centrality, which indicates the number of connections to other nodes in the network and has been used in numerous situations. For example, in the case of protein interactions, proteins with high degree centrality are more likely to be essential than those with low values of degree centrality [21]. Second, we utilized betweenness centrality, which indicates the relevance of a node as capable of holding together communicating nodes: the higher the value the higher the relevance of the node as an organizing regulatory node. The betweenness centrality of a node reflects the amount of control that this node exerts over the interactions of other nodes in the network [22]. Third, we used a double screening scheme (DSS), which combine two algorithms (Maximum Neighborhood Component and Density of Maximum Neighborhood Component) and has been shown to identify hubs that are missed by other algorithms [23].

In general, highly dense modules with low network centralization included many species, all of them with large number of species with high degree centralization and betweenness centrality (Table 1 and Table S1).

### Isolation of the uncultivated organism *Tannerella* sp. OT286

The final set of experiments was designed to demonstrate that the identified modules are biologically meaningful. We decided to show that organisms that have not been cultured yet could be grown based on our results from network analysis.

**Figure 3:**
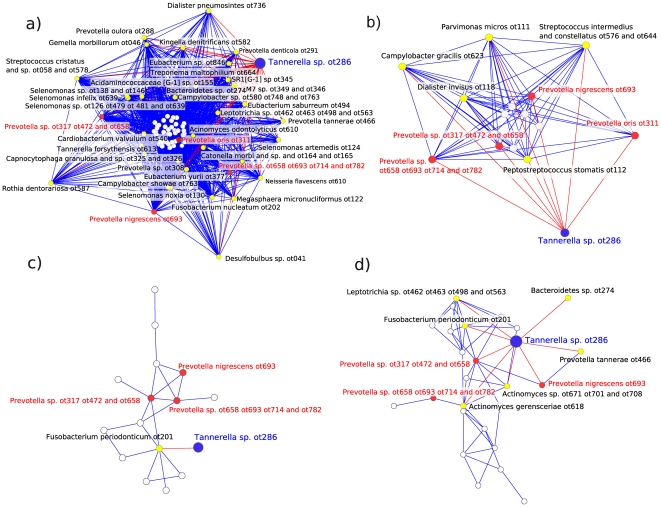
Selecting helpers to isolate the uncultivable organism *Tannerella* sp. OT286. Red edges in the networks show yellow nodes connecting directly to *Tannerella* sp. OT286. The length of the edges is proportional to the strength of the association between species. Oral taxon (OT) for each species/phylotype followed the designation provided in Human Oral Microbiome Database (HOMD) www.homd.org. a) Connections in module turquoise from HOMIM results healthy cluster 1 (51 samples). b) Connections in module red from HOMIM results healthy cluster 2 (37 samples). c) Connections in module grey from HOMIM results from diseased cluster 1 (467 samples). d) Connections in module grey from HOMIM results from diseased cluster 2 (49 samples). In red we show the strains that were tested as helpers in our experiments. Additionally, as negative controls, we tested 2 strains not present in those networks: *Propionibacterium acnes* OT530 and *Lactobacillus casei* OT568.

**Figure 4:**
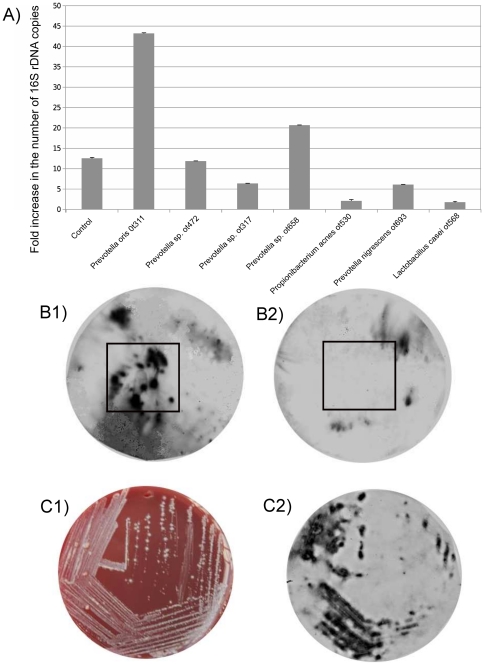
Enrichment and isolation of *Tannerella* sp. OT286. A) qPCR results of the number of 16S rDNA copies of *Tannerella* sp. OT286 after a week of incubation in the presence of different helpers. B1) Results of colony hybridization where the colonies from the initial agar plate enrichment were spread on a plate and a filter paper (black square) was soaked with *Prevotella oris* OT311 and placed on top of the plate. B2) Results of the same experiment but in this case *Lactobacillus casei* OT568, a negative control, was used to soak the filter paper. The black squares indicate where the paper filters were placed soaked with the 2 different species. C1) Streaking isolation of colonies from B1 positive region on agar plates. C2) Colony hybridization of C1 plate showing positively identified *Tannerella* sp. OT286 colonies.

We focused our interest on *Tannerella* sp. OT286, an uncultivated phylotype that has been frequently identified in periodontal health [24] in contrast to its close relative *Tannerella forsythia*, one of the most important periodontal pathogens. To try to isolate this organism we singled out species that were present at least in both clusters from healthy biofilms and if possible had a direct link to *Tannerella* sp. OT286 in the bacterial modules (Fig. 3, Table 2 and Table S2). Moreover, organisms with high centrality would be preferred to those with low centrality and of course we focused on organisms that were culturable. We hypothesized that we could use those organisms as helpers in growing *Tannerella* sp. OT286 from an oral biofilm sample. The selection of helpers to enrich *Tannerella* sp. OT286 was performed as described in the methods section. As shown in Fig. 4a, *Prevotella oris* OT311 and *Prevotella* sp. OT658 increased the growth of *Tannerella* sp. OT286 significantly. Coincidentally, *Prevotella oris* OT311 not only was associated with *Tannerella* sp. OT286 in one of the modules from the healthy biofilms but was also one species with high betweenness centrality. We also observed that *Prevotella oris* OT311 grew by a factor of 19.7 during the period of incubation. Finally, *Propionibacterium acnes* OT530 and *Lactobacillus casei* OT568, which were not present in any of the modules where *Tannerella* sp. OT286 was present had the opposite effect and inhibited its growth (Fig. 4a).

**Table 2:** Species common to the healthy and diseased clusters where *Tannerella* sp. OT286 was also present.

Healthy Cluster 1	Healthy Cluster 2	Diseased Cluster 1	Diseased Cluster 2
Bacteroidetes sp. OT274	Bacteroidetes sp. OT274	Bacteroidetes sp. OT274	Bacteroidetes sp. OT274
Campylobacter gracilis OT623	Campylobacter gracilis OT623		
Dialister invisus OT118	Dialister invisus OT118		
Parvimonas micros OT111	Parvimonas micros OT111		
Prevotella sp. OT317 OT472 OT658	Prevotella sp. OT317 OT472 OT658	Prevotella sp. OT317 OT472 OT658	Prevotella sp. OT317 OT472 OT658
Prevotella sp. OT658 693 714 782	Prevotella sp. OT658 693 714 782	Prevotella sp. OT658 693 714 782	Prevotella sp. OT658 OT693 OT714 OT782
Prevotella nigrescens OT693	Prevotella nigrescens OT693	Prevotella nigrescens OT693	Prevotella nigrescens OT693
Prevotella oris OT311	Prevotella oris OT311		
Prevotella tannerae OT466	Prevotella tannerae OT466	Prevotella tannerae OT466	Prevotella tannerae OT466
Streptococcus sp. OT768 OT767 OT758 OT755 OT745 OT734 OT728 OT721 OT707	Streptococcus sp. OT768 OT767 OT758 OT755 OT745 OT734 OT728 OT721 OT707		
Streptococcus intermedius and anginosus OT543 OT644	Streptococcus intermedius and anginosus OT543 OT644		
Streptococcus intermedius and constellatus OT576 OT644	Streptococcus intermedius and constellatus OT576 OT644		
Streptococcus mitis OT069 OT398	Streptococcus mitis OT069 OT398	Streptococcus mitis OT069 OT398	Streptococcus mitis OT069 OT398

In order to select potential helpers for *Tannerella* sp. OT286 growth we identified organisms that where detected at least in both healthy clusters, whit special emphasis on the organisms that were directly linked to *Tannerella* sp. OT286. OT numbers follow the HOMD nomenclature.

Using *Prevotella oris* OT311 as a helper we first isolated some colonies of *Tannerella* sp. OT286 (Fig. 4b) that were used in a second round of enrichment where the helper and a negative control (*Lactobacillus casei*) were laid on a plate that contained *Tannerella* sp. OT286 from the first isolation. As expected the region of the plate that had been in contact with the helper showed a growth to *Tannerella* sp. OT286 colonies while the same region where the negative control was placed showed no growth of *Tannerella* sp. OT286 (Fig. 4c). As a final control we performed a qPCR on the isolated colonies and found that the number of *Tannerella* sp. OT286 rDNA gene copies was 10^9^ higher in the final suspension, which confirmed that indeed we had finally enriched *Tannerella* sp. OT286. Finally, following a similar procedure isolated colonies were identified on agar plates (Fig. 4c).

## Discussion

In this work, we applied a systems biology approach to simplify the study of complex microbial communities and identity bacterial associations within the community. We used the oral microbial community as a model because dental plaque is a complex biofilm with high level of organization [25]. Around 700 predominant bacterial taxa have been identified in oral cavity [26], [27]. Approximately 35% have not been cultivated and the only information we possess about them is derived from their 16S rRNA phylogenetic affiliation [26], [27]. Additionally we wanted to include periodontal disease samples in our analysis because is one of the most widely studied polymicrobial diseases [28]–[31] and an important environmental perturbation on the composition of the microbial community. Interestingly, the predominant species from diseased sites are different from those found in healthy sites, although the putative pathogens can often be detected in low numbers at normal sites.

Correlation networks were generated using the Weighted Gene Co-expression Network Analysis (WGCNA) [32]. WGCNA analysis is a systems biology method that has been successfully used for describing the expression correlation patterns among genes across microarray samples generating clusters (modules) that are generally related coexpressing metabolic pathways [8], [33]. We decided to apply the same principle to the study of correlation of the abundance of species in the oral biofilm. Species modules could form for a variety of reasons, they may represent physiological or physical species-species interactions or even species that react to similar environmental circumstances. Focusing the analysis on modules (and their intramodular hubs) amounts to a biological data reduction scheme facilitating the study of microbial associations and identification of keystone species within the community. Highly correlated module species are represented and summarized by their first principal component (referred to as the module eigenspecies [34]). The module eigenspecies is used to define measures of module membership which quantify how close a species is to a given module [32].

We first analyzed results from checkerboard DNA-DNA hybridization analysis since it has been extensively used for the study of periodontal disease. Socransky et al. have shown that periodontal bacteria tend to associate in well-defined complexes [20]. These complexes represent bacterial consortia that appear to occur together and that are associated with the biofilms of gingival health, gingivitis and periodontitis. The bacterial modules we obtained agreed with the complexes described by Socransky et al. [20], [35]. When compared with the oral microbial complexes described by Socransky et al. [20] the brown module corresponded to the red complex, the blue module to the yellow complex (*Streptococcus sanguis*. *Streptococcus oralis*, *Streptococcus mitis*, *Streptococcus gordonii* and *Streptococcus intermedius*) and the turquoise module represented a mix of the green complex (*Capnocytophaga* species, *Campylobacter concisus*, *Eikenella corrodens* and *Aggregatibacter actinomycetemcomitans* serotype a.) and the orange complex (*Campylobacter gracilis, Parvimonas micra, Fusobacterium nucleatum, Fusobacterium periodonticum, Prevotella intermedia, Prevotella nigrescens, Campylobacter showae, Campylobacter rectus, Eubacterium nodatum and Streptococcus constellatus*) [20]. The ‘red complex’, which appears later in biofilm development, comprises species that are considered periodontal pathogens, namely, *Porphyromonas gingivalis*, *Treponema denticola*, and *Tannerella forsythia*. Interestingly, from our results *Tannerella forsythia* seems to be the key organism in this module. Accordingly, we found a high correlation of the brown module with clinical traits associated with periodontal disease (Figure S3). However, checkerboard DNA-DNA hybridization is limited to the study of cultivable bacteria and as we mentioned above a large fraction of oral taxa has not been cultivated yet.

The use of HOMIM results improve our knowledge of the architecture of the bacterial associations network in the community since it not only expanded the number of species identified but also included species not-yet-cultivated that could be important in the stability of the community.

We have found two clear defined community structures in health, while in disease it seems there is a singular community highly associated with periodontitis. Interestingly, no consensus networks were identified either between both healthy biofilm samples clusters, which indicates that there is more than one distinct microbial community associated with periodontal health. The factors that determine which of these healthy communities colonize the oral cavity are still unknown. Similarly, no consensus network was obtained for the periodontal samples. However, as mentioned before there is a community that was overwhelmingly identified by both checkerboard DNA-DNA hybridization and HOMIM analysis. Additionally, we could not find consensus network between disease and any of the healthy communities. This observation supports the idea that during disease not only the species present change but also the nature of their interactions.

In general, we found that clusters from healthy samples presented less centralized networks than the disease communities. A very centralized network is dominated by one or a few very central nodes. If these nodes are removed or damaged, the network quickly fragments into unconnected sub-networks. A highly central node can become a single point of failure. A less centralized network has no single points of failure and is more resilient to many environmental challenges. One could hypothesize that modules in healthy communities tend to be more stable than modules in disease communities. Hence, modules in disease communities are controlled by a few number of organisms that could be targeted to altered the community structure.

Once we identified the different networks we were poised to single out the hubs in the community. As mentioned above, identifying hubs is important because they could be targeted to alter the structure of the community to ones favor, either removing hubs associated with disease or promoting the growth of modules linked to health. The idea that there are species in the community that hold special importance in its stability (keystone species) has been used extensively in food webs studies [36]. Recently, Steele et al. have also tried to identify keystone species in microbial ocean food webs [12]. Certain species in complex microbial communities may play the role of keystone species by maintaining a stable and functional community, as is the case of *Bacteroides thetaiotaomicron* in the gut microbiota [37]. Given the low centrality of most of the modules identified, degree centrality (indicates the number of connections to other nodes in the network) and betweenness centrality (indicates the relevance of a node as capable of holding together communicating nodes) in most cases identified a large number of species as important, though they generally agreed in which ones were hubs. In those cases the Double Screening Scheme (DSS) identified lower number of species as important in holding the network together. However, DSS did not usually agree with other centralities. The importance of the identified hubs should be tested in the laboratory but by using this kind of analysis we have targeted specific species as potentially important, which greatly simplify the analysis of the microbial community.

We have provided an empirical evidence of the accuracy of this kind of analysis by isolating a not-yet-cultivated organism (*Tannerella sp.* OT286) based on the network analysis results. Kaeberlein et al. demonstrated that “uncultivable” organisms that did not grow in artificial media alone formed colonies in the presence of other organisms, and they proposed that this observation may explain the uncultivability of certain species in the laboratory [38]. Borrowing this idea we selected helper organisms that enriched *Tannerella* sp. OT286 when incubating plaque and saliva in an artificial saliva medium. This is a poor medium that does not allow an overgrowth of fast growing organisms. Two of the strains tested significantly enriched *Tannerella* sp. OT286 (*Prevotella oris* OT311 and *Prevotella* sp. OT658). *Prevotella* sp. OT658 is part of 2 of the *Prevotella* clusters that were identified as associated with *Tannerella* sp. OT286. Both, *Prevotella oris* OT311 and *Prevotella* sp. OT658, had high centrality and were present at least in the modules from health where *Tannerella* sp. OT286 appeared (Fig. 3). Interestingly, the 2 strains tested as negative controls that were never identified as associated with *Tannerella* sp. OT286 in the bacterial modules (*Propionibacterium acnes* OT530 and *Lactobacillus casei* OT568) had an inhibitory effect on its growth (Fig. 4a).

These results provide direct evidence that network analysis on complex microbial communities where there is a cooperative environment is a useful tool to derive hypotheses that can be tested in the laboratory. We have shown how a not-yet-cultivated oral species was cultivated based on the results obtained using systems biology methods applied to microbial communities. We believe that the same principle could be used to specifically target hubs in the modules or to selectively increase growth of modules related to health.

## Methods

### Samples, Checkerboard analysis and Human Oral Microbe Identification Microarray (HOMIM)

Checkerboard results from 2,565 individual subgingival plaque samples from patients with periodontitis were used for analysis. Checkerboard was performed as described elsewhere [15]. Briefly, denatured DNA from the samples was fixed in separate lanes on a single membrane mounted in a Miniblotter 45. The membrane was then rotated 90 degrees in the same device, which enabled simultaneous hybridization with the different DNA probes. A MiniSlot device allowed lysates loaded in parallel channels to be aspirated through the membrane, depositing horizontal lanes on the membrane surface. Hybridizations were performed in vertical lanes with either digoxigenin-labeled whole genomic probes or 16S rRNA-based oligonucleotide probes directly conjugated to alkaline phosphatase.

Human Oral Microbe Identification Microarray (HOMIM) used on those experiments detected a total of 276 species of oral bacteria. Samples and procedures for HOMIM are described elsewhere [3]. Briefly, 16S rRNA-based, reverse-capture oligonucleotide probes (typically 18 to 20 bases) were printed on aldehyde-coated glass slides. Subject sample 16S rRNA genes were PCR amplified from DNA extracts using 16S rRNA universal forward and reverse primers and labeled via incorporation of Cy3-dCTP in a second nested PCR. The labeled 16S amplicons were hybridized overnight to probes on the slides. After washing, the microarray slides were scanned using an Axon 4000B scanner and crude data was extracted using GenePix Pro software. A total of 89 microarrays from healthy subgingival sites and 514 subgingival sites from individuals with periodontitis were used for network analysis.

### Bayesian Principal Component Analysis (BPCA) Missing Value Estimator

To estimate missed values in the arrays we used the bpca[17] script in R. The script is a port of the Matlab version provided by Shigeyuki Oba [17] and it is included in the pcaMethods R package. Before BPCA analysis, all values of fluorescence were normalized against the values of fluorescence of a 16S rRNA universal probe in the array. For the analysis we computed the average fluorescence of all probes for each specific bacterial species.

### Correlation Network Analysis

WGCNA [32] starts by calculating a correlation matrix containing all pairwise Pearson correlations between all probe sets across all subjects. We define correlation networks as undirected, weighted species networks. The nodes of such a network correspond to species and edges between species are determined by the pairwise Pearson correlations between species. The first step in the analysis is identifying outlier samples using absolute hierarchical cluster analysis. After removing the outliers for analysis we would construct a weighted network choosing a thresholding power β to which co-occurrence similarity is raised to calculate adjacency [39]. Instead of focusing on the significance of the correlation Zhang and Horvath have proposed to choose the soft thresholding power based on the criterion of approximate scale-free topology [39]. By raising the absolute value of the Pearson correlation to a power β≥1 (soft thresholding), the weighted species co-expression network construction emphasizes large correlations at the expense of low correlations. Specifically, aij = |cor(xi, xj)|^β^ represents the adjacency of an (unsigned) weighted species co-express network. We used the scale free topology criterion to choose the soft threshold. The choice of the power has an effect on the scale fitting index and it has to be selected so that approximate scale free fit can be achieved [39]. To minimize spurious associations during module identification we transformed the adjacency into Topological Overlap Matrix and calculate the corresponding dissimilarity. Species are organized into modules, using this topological overlap measure as a robust measure of interconnectedness in a hierarchical cluster analysis [40], [41]. We used average linkage hierarchical clustering to construct the corresponding dendrogram. Module identification amounts to the identification of individual branches with a certain number of species. Finally, data network was exported to be visualized using Cytoscape [42]. To relate modules to clinical traits we also used WGCNA package [32] correlating the eigengene for each module with the traits of interest and look for significant associations based on their p-values.

### Global parameters describing networks

Global descriptors of the modules were obtained using Cytoscape [42]. The neighborhood of a given node n is the set of its neighbors. The connectivity is the size of its neighborhood. The average number of neighbors indicates the average connectivity of a node in the network. A normalized version of this parameter is the network density. Density ranges between 0 and 1. It shows how densely the network is populated with edges, A network which contains no edges and solely isolated nodes has a density of 0. In contrast, the density of a clique is 1. Another related parameter is the network centralization [43]. Networks whose topologies resemble a star have a centralization close to 1, whereas decentralized networks are characterized by having a centralization close to 0.

In undirected networks, the clustering coefficient C_n_ of a node n is defined as C_n_ = 2e_n_/(k_n_(k_n_−1)), where k_n_ is the number of neighbors of n and e_n_ is the number of connected pairs between all neighbors of the network [44], [45]. The clustering coefficient of a node is always a number between 0 and 1. The network clustering coefficient is the average of the clustering coefficients for all nodes in the network. Nodes with less than two neighbors are assumed to have a clustering coefficient of 0.

### Network centralities

We then determined network centralities on the modules obtained from network analysis. Centralities were assessed using Cytoscape [42] and the plugin CytoHubba v1.1 [23]. We calculated Degree centrality and Betweenness centrality using Cytoscape and the double screening scheme (DSS) of Maximum Neighborhood Component (MNC) and Density of Maximum Neighborhood Component (DMNC) using CytoHubba v.1.1.

### Selection of helpers to enrich *Tannerella* sp. OT286

Oral taxon (OT) for each species/phylotype followed the designation provided in Human Oral Microbiome Database (HOMD) www.homd.org. Helpers were selected following several criteria. First, we were limited to using only cultivable species in the modules. Second, we selected only species that were associated with *Tannerella* sp. OT286 in all modules (Figure 3). Finally, we focused our interest specially on species directly associated with *Tannerella* sp. OT286 in the healthy modules, since it has been described as present mainly in healthy individuals. Samples of saliva and dental plaque were inoculated in 10 ml of artificial saliva medium with high concentrations of mucin [46], pre-reduced in an anaerobic chamber for 24 h. Helper strains grown on Tripticase Soy Agar (BBL) supplemented with 20% sheep blood and 5 gr/l of Yeast extract for 24 h and resuspended in artificial saliva medium at a turbidity of MacFarlan 3 (approximately 10^8^ CFU/ml). Finally, 1 ml of each suspension was added to 1 ml of saliva-dental plaque in artificial saliva medium. No bacteria were added to control set and all tubes were incubated anaerobically for 7 days at 37°C. The concentration of *Tannerella* sp. OT286 was measure by qPCR. Total chromosomal DNA was isolated from 1 ml of each set by UltraClean® Microbial DNA Isolation Kit (Mo Bio Laboratories, Inc). All measurements were performed by triplicate. 20 ng of DNA, in all cases, were subjected to qPCR using an iCycler 584BR (Bio-Rad Laboratories) with Taqman Prime Assays (IDT DNA technologies), and Taqman Gene Expression Master Mix (Applied Biosystems). Primers and probes used for measuring *Tannerella* sp. OT286 16S rDNA copy numbers were: 5′- Probe:/56-FAM/TGCATCCGA/ZEN/TCGCTCGGT/3IABkFQ/-3′; Primer1: 5′–CGGCCCTTACATCCGGGGCG-3′ and Primer 2: 5′- CCGATCCGAACTGAGACAGGG -3′ designed by Züger [24]. For *Prevotella oris* OT311 we used: Probe 5′-/56-FAM/GAATTGCAG/ZEN/GCGAAGGCTTCAG/3IABkFQ/-3′; Primer 1: 5′–AACCATGCAGCACCTTCACAGA -3′ and Primer 2: 5′- TTCGATGATACGCGAGGAACCT- 3′. They were designed with ARB [47]. In all cases Taqman probes were labeled at the 5′ end with FAM reporter dye and labeled at the 3′end with the quencher dye Iowa Black ™ FQ. PCR conditions included denaturation at 95°C for 15 minutes, and then 40 cycles of 95°C for 30 seconds, 62°C for 1 minute, and 72°C for 30 seconds, followed by melting curve analysis. Fluorescence data was captured during annealing reactions, and specificity of the amplification was confirmed using melting curve analysis. Data were collected and recorded by iCycler iQ software (Bio-Rad Laboratories) and initially determined as a function of threshold cycle (C_t_). C_t_ was defined as the cycle at which the fluorescence intensity in a given reaction tube rose above background, which was calculated as 10 times the mean standard deviation (SD) of fluorescence in all wells over the baseline cycles. Levels of increased 16S rDNA copies were expressed relative to control levels, calculated as 2^Δ−[Ctexp−Ctcontrol)^ [48].

### Enrichment of the uncultivated organism *Tannerella* sp. OT286

Enrichment followed a two step procedure. First, saliva and dental plaque samples were spread on *Tannerella forsythia* agar (ATCC 1921-NAM agar plate) previously inoculated with 1 ml of suspension of the “helper” strain *Prevotella oris* OT311 at 108 CFU/ml. After 7 days of anaerobic incubation at 37°C, a dry Nylon membrane positively charged (Roche) was placed on plate for 10 min and colony hybridization was done [49]. The transferred membrane was 30 minutes blocked for unspecific binding at 55°C in blocking buffer (Roche) and 40 ng/ml of DIG labeled probe (5′-TGCATCCGATCGCTCGGT/3 DIG_N/3′) [24] were hybridized on blocking buffer at 65°C for 3 h. Wash and develop blot under same conditions as with DIG labeling Kit (Roche). Plates were incubated for an additional 7 days after transferring membranes. A second enrichment of primary cultivated *Tannerella* sp. OT286 was done by spreading those colonies resuspended in 500 ml of *Tannerella forsythia* broth (ATCC). Sterile filters were soaked on a suspension of the “helper” strain *Prevotella oris* OT311 and control strain *Lactobacillus casei* ATCC 334, both at 108 CFU/ml and placed on the middle of the plate previously inoculated with *Tannerella* sp. OT286. Seven days of anaerobic incubation were followed and colony hybridization was done as described above.

## Supporting Information

Figure S1 **Identification of outlier checkerboard DNA-DNA hybridization and HOMIM samples by hierarchical Clustering based on the array profiles.** a) Samples used for checkerboard DNA-DNA hybridization analysis, all of them were obtained with individuals with periodontal disease. b) Samples from healthy individuals used in HOMIM analysis. Significantly different sample clusters are grouped inside a rectangles (Cluster 1 blue, Cluster 2 green). c) Samples from individuals with periodontal disease used in HOMIM analysis. Significantly different sample clusters are grouped inside a rectangles (Cluster 1 blue, Cluster 2 green). Outliers are indicated in red.
(PDF)

Figure S2 **Heat maps showing the abundance of the different species across samples.** WGCNA analysis allows visualize changes in abundance of species across samples. Red represent high abundance while green represent low abundance. The order of species is the same in the 4 pictures. a) Heat-map of species abundance across samples in healthy Cluster 1. b) Heat-map of species abundance across samples in healthy Cluster 2. c) Heat-map of species abundance across samples in disease Cluster 1. d)Heat-map of species abundance across samples in disease Cluster 2.
(PDF)

Figure S3 **Module-trait associations.** WGCNA analysis allows to assess the importance of module on a specific clinical trait. In the present figure each row corresponds to a module eigengene, column to a trait. Each cell contains the corresponding correlation and p-value. The table is color-coded by correlation according to the color legend. Plaque: plaque index indicating the level of accumulated biofilm, Red: gingival redness, BOP: bleeding on probing, Sup: suppuration, PD: pocket depth, AL: attachment level, NMT: number of missing teeth, redcomplexEn.cnts: counts of *Porphyromonas gingivalis*, *Treponema denticola*, *Tannerella forsythia* and *Eubacterium nodatum*, BPD: baseline pocket depth.
(PDF)

Table S1 **Network centralities for the detected modules.** Species with high centralities measured by different algorithms. These species could be considered important ‘hubs’ in the different modules. Degree centrality indicates the number of connections to other nodes in the network. Betweenness centrality of a node indicates its relevance as capable of holding together communicating nodes. DSS stands for Double Screening Scheme and combines the use of Maximum Neighborhood Component (MNC) and Density of Maximum Neighborhood Component (DMNC) and has been shown to identify hubs that are missed by other algorithms.
(DOC)

Table S2 **Species nodes directly connected to** ***Tannerella*** **sp. OT286.** These are the bacterial species whose edges were directly connected to *Tannerella* sp. OT286 in the 3 sample clusters analyzed, two from healthy sites and 1 from diseased sites.
(DOC)
