# Supplementary figures and images for: Correlation Network Analysis Applied to Complex Biofilm Communities
Source: PLoS One. 2011 Dec 7;6(12):e28438. doi: 10.1371/journal.pone.0028438 (PMC3233593; doi:10.1371/journal.pone.0028438)

CLUSTER DENDROGRAM

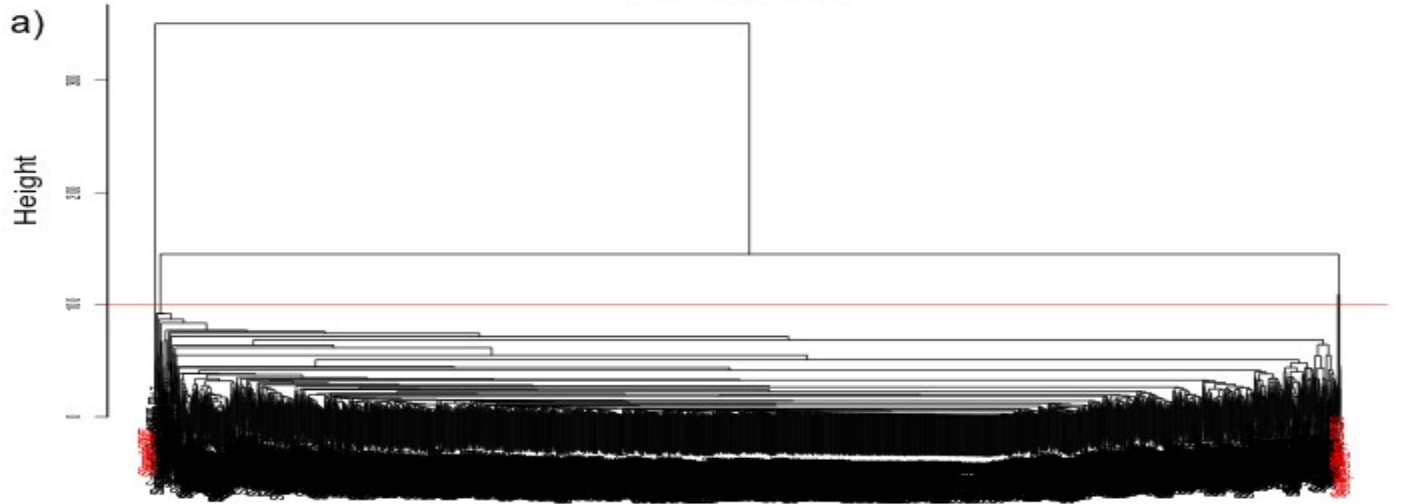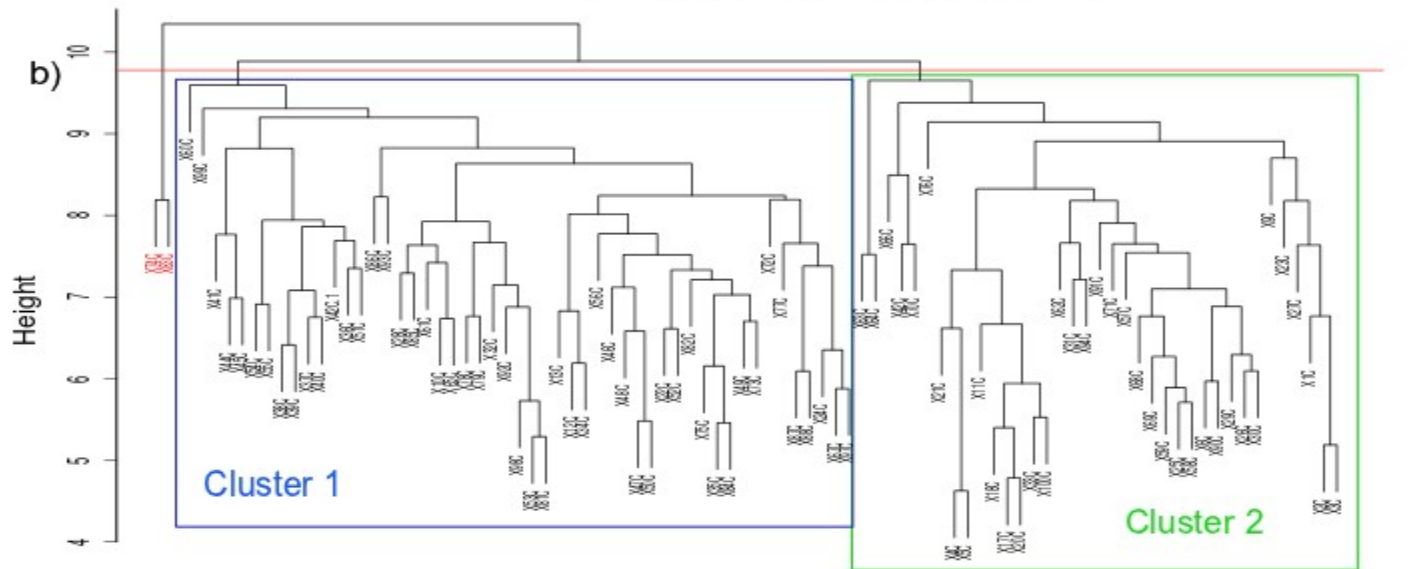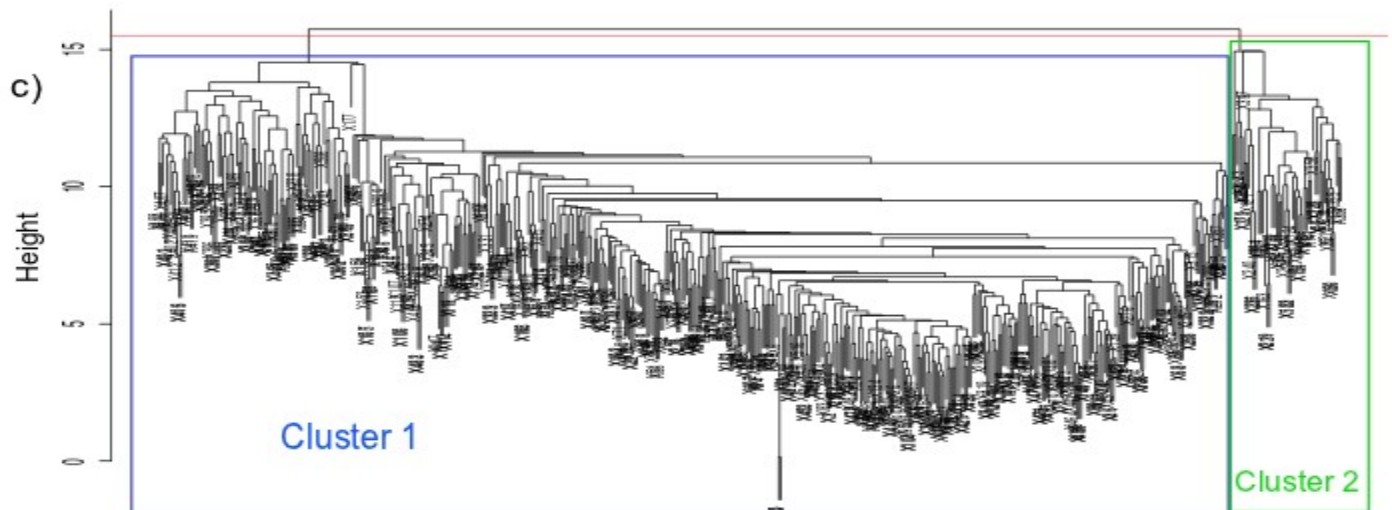

Supplement: Figure S1 — Identification of outlier checkerboard DNA-DNA hybridization and HOMIM samples by hierarchical Clustering based on the array profiles. a) Samples used for checkerboard DNA-DNA hybridization analysis, all of them were obtained with individuals with periodontal disease. b) Samples from healthy individuals used in HOMIM analysis. Significantly different sample clusters are grouped inside a rectangles (Cluster 1 blue, Cluster 2 green). c) Samples from individuals with periodontal disease used in HOMIM analysis. Significantly different sample clusters are grouped inside a rectangles (Cluster 1 blue, Cluster 2 green). Outliers are indicated in red. (PDF) [file pone.0028438.s001.pdf]

a) Bacterial species

Samples

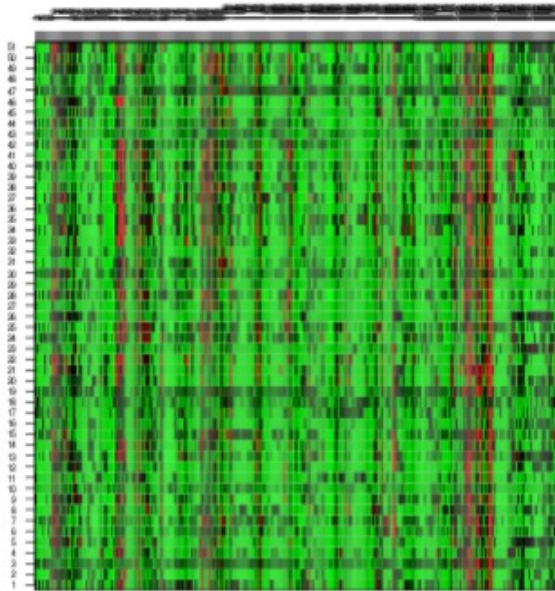

b) Bacterial species

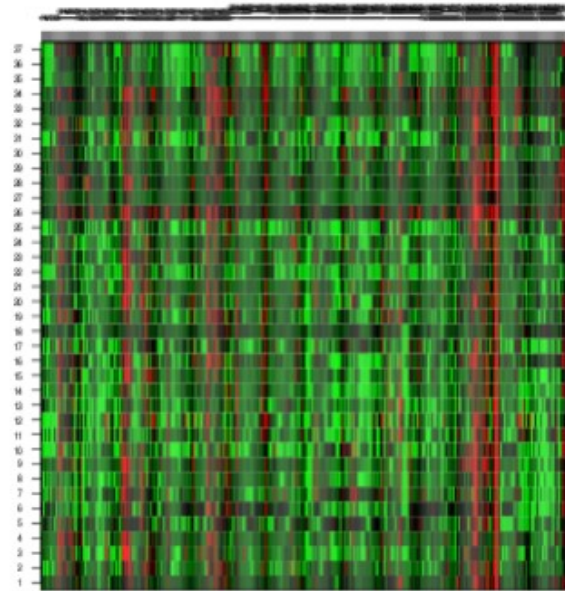

c)

Samples

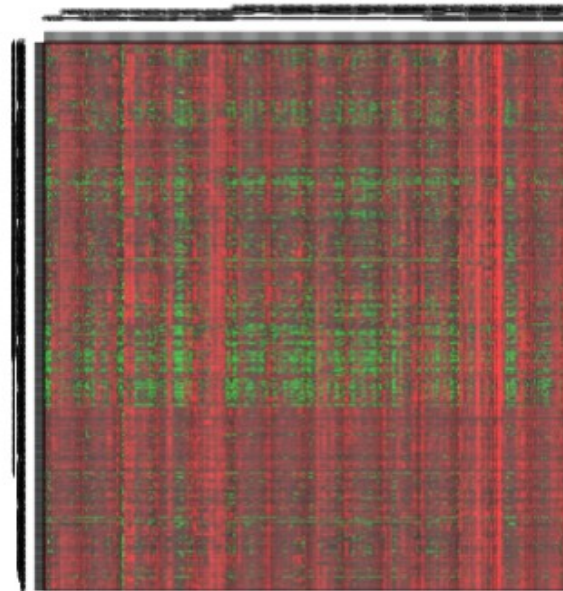

d)

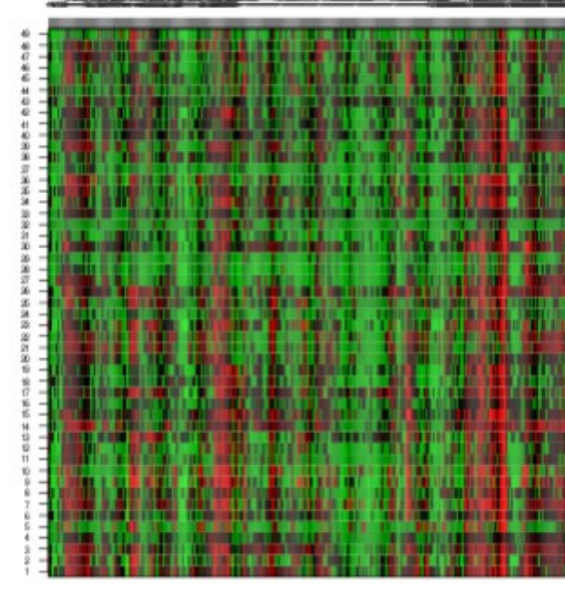

Supplement: Figure S2 — Heat maps showing the abundance of the different species across samples. WGCNA analysis allows visualize changes in abundance of species across samples. Red represent high abundance while green represent low abundance. The order of species is the same in the 4 pictures. a) Heat-map of species abundance across samples in healthy Cluster 1. b) Heat-map of species abundance across samples in healthy Cluster 2. c) Heat-map of species abundance across samples in disease Cluster 1. d)Heat-map of species abundance across samples in disease Cluster 2. (PDF) [file pone.0028438.s002.pdf]

## Module-trait relationships

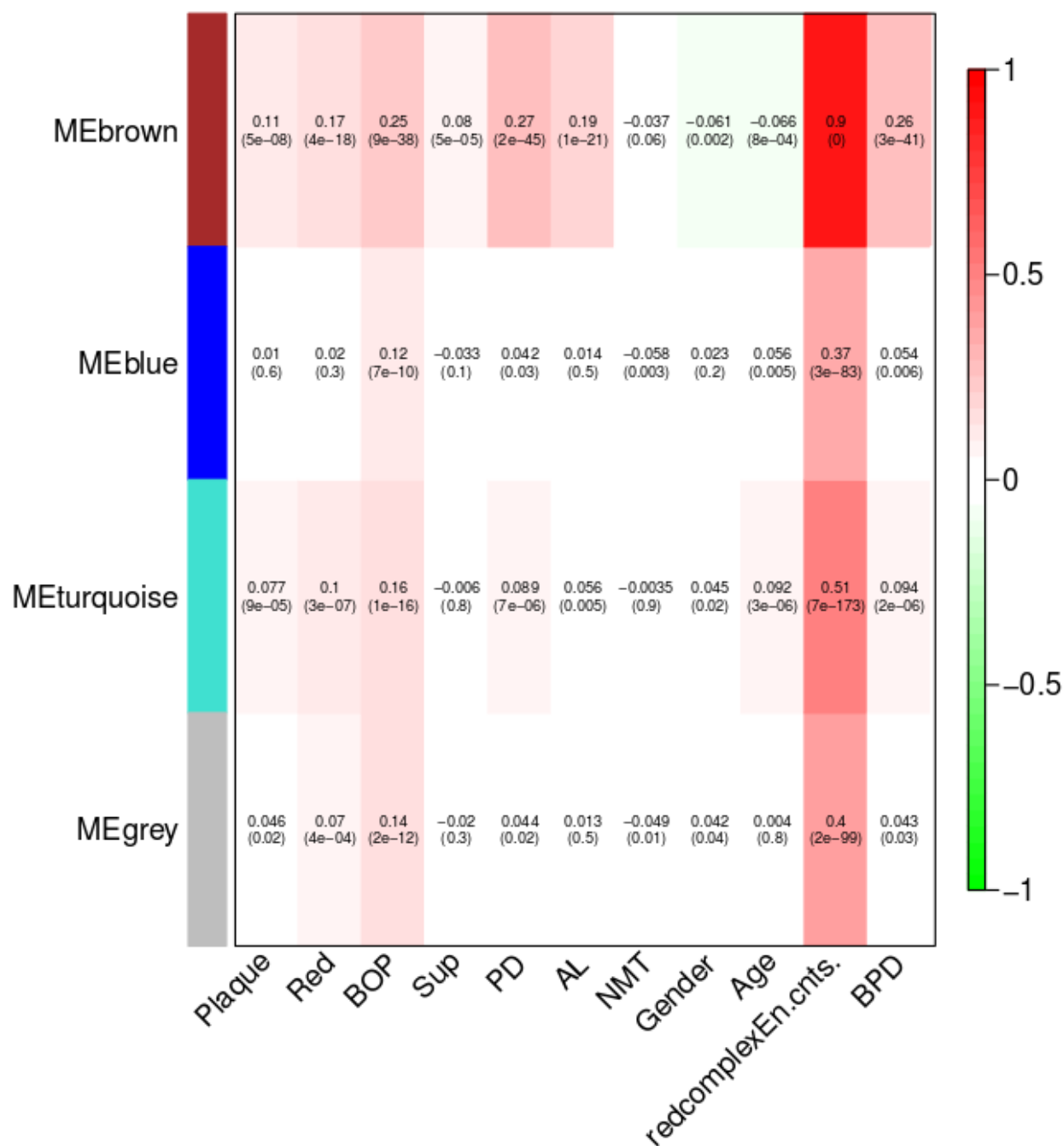

Supplement: Figure S3 — Module-trait associations. WGCNA analysis allows to assess the importance of module on a specific clinical trait. In the present figure each row corresponds to a module eigengene, column to a trait. Each cell contains the corresponding correlation and p-value. The table is color-coded by correlation according to the color legend. Plaque: plaque index indicating the level of accumulated biofilm, Red: gingival redness, BOP: bleeding on probing, Sup: suppuration, PD: pocket depth, AL: attachment level, NMT: number of missing teeth, redcomplexEn.cnts: counts of Porphyromonas gingivalis, Treponema denticola, Tannerella forsythia and Eubacterium nodatum, BPD: baseline pocket depth. (PDF) [file pone.0028438.s003.pdf]
