# Supplementary material for: Correlation Network Analysis Applied to Complex Biofilm Communities
Source: PLoS One. 2011 Dec 7;6(12):e28438. doi: 10.1371/journal.pone.0028438 (PMC3233593; doi:10.1371/journal.pone.0028438)
Supplement: Table S1 — Network centralities for the detected modules. Species with high centralities measured by different algorithms. These species could be considered important ‘hubs’ in the different modules. Degree centrality indicates the number of connections to other nodes in the network. Betweenness centrality of a node indicates its relevance as capable of holding together communicating nodes. DSS stands for Double Screening Scheme and combines the use of Maximum Neighborhood Component (MNC) and Density of Maximum Neighborhood Component (DMNC) and has been shown to identify hubs that are missed by other algorithms. (DOC) [file pone.0028438.s004.doc]

| **Sample** | **Degree** | **Betweenness** | **DSS** |
| --- | --- | --- | --- |
| **Checkerboard** |  |  |  |
| Module brown | *Tannerella forsythia* | *Tannerella forsythia* | *Tannerella forsythia* |
| Module grey | *Prevotella melaninogenica* | *Prevotella melaninogenica* | *Prevotella melaninogenica* |
| Module turquoise | *Prevotella nigrescens* | *Prevotella nigrescens* | *Prevotella nigrescens* |
| Module blue | *Streptococcus anginosus* | *Streptococcus anginosus* | *Eubacterium saburreum* |

| **Sample** |  |  |  |
| --- | --- | --- | --- |
| **HOMIM Healthy** | **Degree** | **Betweenness** | **DSS** |
| **Cluster 1** |  |  |  |
| Module Blue | Oribacterium sp. OT078  Selenomonas sp. OT136 149 478  Peptostreptococcaceae sp. OT106  Lactobacillus vaginalis OT051  Lactobacillus salivarius OT756  Lachnospiraceae sp. DO016 OT079  Selenomonas sp. OT126  Acidaminococcaceae[G-2] sp. OT131  Selenomonas sp. OT133  Burkholderia sp. OT406 | Peptostreptococcaceae sp. OT106  Selenomonas sp. OT136 149 478  Lactobacillus vaginalis OT051  Lachnospiraceae sp. DO016 OT079  Selenomonas sp. OT126  Acidaminococcaceae[G-2] sp. OT131  Selenomonas sp. OT133  Burkholderia sp. OT406 | Lachnospiraceae G3 sp. OT096  Lachnospiraceae OT078 372 |
| Module Brown | Treponema sp. OT257  Scardovia inopinata OT642 | Treponema socranskii OT769  Scardovia inopinata OT642 | Olsenella uli OT038  Slackia exigua OT682 |
| Module Green | Mycoplasma faucium OT606  Streptococcus downei OT594 | Mycoplasma faucium OT606  Streptococcus downei OT594 | Shuttleworthia satelles OT095  Streptococcus salivarius OT755 |
| Module Grey | Actinomyces sp. OT169  Atopobium parvulum OT723  Streptococcus australis OT073  Atopobium rimae OT754  Corynebacterium durum OT595  Actinomyces odontolyticus and meyeri OT671 701 | Corynebacterium durum OT595  Haemophilus parainfluenzae OT718 | Actinomyces sp. OT169  Corynebacterium durum OT595  Actinomyces odontolyticus and meyeri OT671 701 |
| Module Turquoise | Campylobacter showae OT763  Catonella morbi and sp. OT164 165  Selenomonas infelix OT639  Prevotella nigrescens OT693 | Campylobacter showae OT763  Catonella morbi and sp. OT164 165  Selenomonas sp. OT138 146  Selenomonas infelix OT639  Prevotella nigrescens OT693 | Tannerella forsythensis OT613  Eikenella corrodens OT577 |
| Module Yellow | Prevotella sp. OT298  Porphyromonas sp. OT279  Prevotella mutisaccharivorax OT794  Stenotrophomonas maltophilia OT663 | Prevotella sp. OT298  Prevotella pallens and sp. OT310 714  Porphyromonas sp. OT279  Prevotella mutisaccharivorax OT794  Stenotrophomonas maltophilia OT663 | Prevotella sp. OT658 693 782 714  Capnocytophaga sp. OT332  Porphyromonas catoniae OT283 |

| **Sample** |  |  |  |
| --- | --- | --- | --- |
| **HOMIM Healthy** | **Degree** | **Betweenness** | **DSS** |
| **Cluster 2** |  |  |  |
| Module Black | Bifidobacteriaceae Family sp. OT198 407 586 588  Bifidobacterium dentium OT588  Streptococcus mutans OT686 | Bifidobacteriaceae Family sp. OT198 407 586 588  Treponema socranskii and sp. OT268 769  Bifidobacterium dentium OT588 | Streptococcus anginosus OT543  Streptococcus sobrinus OT768 |
| Module Blue | Ochrobactrum anthropi OT544  Prevotella mutisaccharivorax OT794  Capnocytophaga sp. OT336  Prevotella sp. DO027 OT306  Capnocytophaga sp. OT332  Prevotella pallens and sp. OT310 714  Prevotella buccae OT560  Prevotella oralis OT705 | Capnocytophaga sp. OT332  Prevotella pallens and sp. OT310 714  Prevotella oralis OT705 | Cardiobacterium hominis and valvulum OT540 633  Prevotella intermedia OT643 |
| Module Brown | Acidaminococcaceae G1 sp. OT135 148  Selenomonas sp. OT149 481  Selenomonas sp. OT146  Selenomonas flueggii OT125  Selenomonas sp. OT133  Enterococcus faecalis OT604 | Veillonella atypica OT524  Enterococcus faecalis OT604 | Selenomonas sp. OT129 150  Neisseria sp. OT011 014 598 |
| Module Green | Selenomonas sp. OT138 146  Solobacterium moorei OT678  Selenomonas sp. OT126 479 481 639  Catonella morbi and sp. OT164 165  Selenomonas infelix OT639 | Leptotrichia sp. OT417  Selenomonas sp. OT138 146  Leptotrichia sp. OT462  Solobacterium moorei OT678  Selenomonas sp. OT126 479 481 639  Catonella morbi and sp. OT164 165  Selenomonas infelix OT639 | Fusobacterium nucleatum ss polymorphum OT202 |
| Module Grey | Haemophilus parainfluenzae OT718  Streptococcus sanguinis OT758  Capnocytophaga sp. OT335  Lautropia mirabilis OT022  Capnocytophaga sp. OT326  Streptococcus oralis OT707  Actinomyces sp. OT179 | Haemophilus parainfluenzae OT718  Kingella oralis OT706  Lautropia mirabilis OT022 | Streptococcus infantis and cristatus OT578 638  Streptococcus sanguinis OT758  Lautropia mirabilis OT022  Actinomyces sp. OT179 |
| Module Magenta | Staphylococcus aureus OT550  Lactobacillus vaginalis OT051  Mogibacterium tidmidum OT042  Lactobacillus sp. OT568 716 749  Lachnospiraceae OT078 372  Lactobacillus sp. OT461 | Staphylococcus aureus OT550  Eubacterium minutum sp. OT673  Lactobacillus vaginalis OT051  Mogibacterium tidmidum OT042  Lactobacillus sp. OT568 716 749  Lachnospiraceae OT078 372  Lactobacillus sp. OT461 | Mycoplasma faucium OT606  Mycoplasma hominis OT632 |
| Module Pink | Synergistes sp. OT362  Synergistes sp. OT358  Synergistes sp. OT357  Eubacterium [G-1] sulci OT467  Streptococcus downei OT594 | Capnocytophaga granulosa and sp. OT325 326  Eubacterium [G-1] sulci OT467  Streptococcus downei OT594 | Synergistes sp. OT360 362 453  Lactobacillus salivarius OT756  Shuttleworthia satelles OT095 |
| Module Red | Parvimonas micros OT111  Prevotella nigrescens OT693  Dialister invisus OT118 | Parvimonas micros OT111  Prevotella nigrescens OT693  Dialister invisus OT118 | Bacteroidetes sp. OT274  Prevotella oris OT311  Prevotella sp. OT317 472 658 |
| Module Turquoise | Olsenella uli and sp. OT038 807  Actinomyces sp. OT176  Scardovia sp. OT195 | Treponema vincentii OT029  Actinomyces sp. OT176  Actinomyces viscosus OT688 | Lactobacillus fermentum OT608  Treponema medium OT667  Atopobium rimae OT754 |
| Module Yellow | Eubacterium OT846  Helicobacter pylori OT812  Abiotrophia defectiva OT389  Eikenella corrodens OT577  Lachnospiraceae G3 sp. OT096  Clostridiales F2 G1 sp. OT075  Burkholderia sp. OT406  Eubacterium nodatum OT694  Peptostreptococcaceae [G-4] sp. OT103 369  Lachnospiraceae sp. OT079 | Eubacterium OT846  Helicobacter pylori OT812  Abiotrophia defectiva OT389  Eikenella corrodens OT577  Lachnospiraceae G3 sp. OT096  Clostridiales F2 G1 sp. OT075  Burkholderia sp. OT406  Eubacterium nodatum OT694  Peptostreptococcaceae [G-4] sp. OT103 369  Lachnospiraceae sp. OT079 | Eubacterium saphenum OT759  Eubacterium sp. OT081  Lachnospiraceae[G-5] sp. OT080  Desulfobulbus sp. OT041 |

| **Sample** |  |  |  |
| --- | --- | --- | --- |
| **HOMIM Disease** | **Degree** | **Betweenness** | **DSS** |
| **Cluster 1** |  |  |  |
| Module Blue | Actinomyces sp. OT169  Lactobacillus fermentum OT608  Lactobacillus acidophilus OT529 | Corynebacterium durum OT595  Actinomyces sp. OT169  Lactobacillus acidophilus OT529 | Streptococcus australis OT065 073  Cryptobacterium curtum OT579 |
| Module Green | Peptostreptococcus stomatis OT112  Pseudoramibacter alactolyticus OT538  Mogibacterium tidmidum OT042 | Peptostreptococcus stomatis OT112  Pseudoramibacter alactolyticus OT538  Mogibacterium tidmidum OT042 | Tannerella forsythensis OT613 X56  Eubacterium nodatum OT694 |
| Module Grey* | Fusobacterium periodonticum OT201  Leptotrichia sp. OT462 463 498 563  Prevotella sp. OT658 693 714 782  Prevotella sp. OT317 472 658  Prevotella tannerae OT466 | Prevotella tannerae OT466  Prevotella sp. OT317 472 658  Prevotella sp. OT658 693 714 782  Fusobacterium periodonticum OT201 | Prevotella tannerae OT466  Prevotella sp. OT658 693 714 782  Prevotella nigrescens OT693 |
| Module Red | Selenomonas noxia OT130  Selenomonas dianae OT139  Selenomonas sp. OT126 479 481 639 | Selenomonas noxia OT130  Selenomonas flueggii OT125  Selenomonas dianae OT139 | Selenomonas sputigena OT151  Selenomonas sp. OT134 |
| Module Turquoise | Capnocytophaga sp. OT324  Porphyromonas catoniae OT283  Burkholderia cepacia OT571  Bacteroides heparinolyticus OT784  Prevotella buccae OT560  Burkholderia sp. OT406 | Capnocytophaga sp. OT329  Capnocytophaga sp. OT324  Porphyromonas catoniae OT283 | Aggregatibacter actinomycetemcomitans OT531  Prevotella sp. OT298 |
| Module Yellow | Streptococcus sp. OT071 755 758  Streptococcus oralis OT707  Streptococcus sp. OT768 767 758 755 745 734 728 721 707 | Streptococcus sp. OT071 755 758  Streptococcus oralis OT707  Streptococcus sp. OT768 767 758 755 745 734 728 721 707 | Streptococcus sanguinis OT758  Streptococcus australis OT073 |

| **Sample** |  |  |  |
| --- | --- | --- | --- |
| **HOMIM** | **Degree** | **Betweenness** | **DSS** |
| **Disease 2** |  |  |  |
| Module Blue | Lactobacillus gasseri and johnsonii OT 615 819  Lactobacillus fermentum OT608  Streptococcus sobrinus OT768 | Lactococcus lactis OT804  Lactobacillus gasseri and johnsonii OT 615 819  Streptococcus sobrinus OT768 | Fusobacteria sp. OT210 220  Synergistes sp. OT359 |
| Module Brown | Scardovia sp. OT195  Actinomyces sp. OT445 448  Treponema sp. OT257  Treponema lecithinolyticum OT653 | Actinomyces naeslundii OT176  Treponema sp. OT231  Scardovia sp. OT195 | Actinomyces sp. OT175  Actinomyces odontolyticus and meyeri OT671 701 |
| Module Green | Streptococcus constellatus OT576  Treponema sp. OT262 264 265 541  Synergistes sp. OT358 361 362 363 453 452  Filifactor alocis OT539  TM7 sp. OT346 | Filifactor alocis OT539  Treponema sp. OT262 264 265 541  TM7 G-1 sp. OT349 346 | Streptococcus constellatus OT576  Synergistes sp. OT358 361 362 363 453 452  Fusobacterium naviforme OT200 |
| Module Grey | Prevotella sp. OT317 472 658  Actinomyces odontolyticus OT701  Fusobacterium periodonticum OT201  Tannerella sp. OT286  Leptotrichia sp. OT462 463 498 563 | Actinomyces gerensceriae OT618  Actinomyces odontolyticus OT701  Prevotella sp. OT317 472 658 | Streptococcus salivarius and sp. OT067 755  Streptococcus mitis bv2 and sp. OT069 398 |
| Module Red | Selenomonas sp. OT126 479 481 639  Campylobacter gracilis OT623  Selenomonas sp. OT138 146 | Selenomonas sp. OT126 479 481 639  Campylobacter gracilis OT623  Selenomonas sp. OT138 146 | Selenomonas noxia OT130  Selenomonas infelix OT639 |
| Module Turquoise | Eikenella corrodens OT577  Capnocytophaga sp. OT324  Prevotella sp. OT376  Megasphaera sp. OT123 | Eikenella corrodens OT577  Bacteroides heparinolyticus OT784  Capnocytophaga ochracea OT700  Capnocytophaga sp. OT324 | Prevotella OT473 474  Lachnospiraceae G3 sp. OT096 |
| Module Yellow | Streptococcus intermedius and anginosis OT543 644  Streptococcus sp. OT768 767 758 755 745 734 728 721 707  Streptococcus sp. OT071 755 758  Streptococcus oralis OT707 | Streptococcus intermedius and anginosis OT543 644  Treponema denticola OT584  Streptococcus sp. OT768 767 758 755 745 734 728 721 707 | Streptococcus sanguinis OT758  Streptococcus infantis and cristatus OT578 638 |
