# Supplementary material for: Correlation Network Analysis Applied to Complex Biofilm Communities
Source: PLoS One. 2011 Dec 7;6(12):e28438. doi: 10.1371/journal.pone.0028438 (PMC3233593; doi:10.1371/journal.pone.0028438)
Supplement: Table S2 — Species nodes directly connected to Tannerella sp. OT286. These are the bacterial species whose edges were directly connected to Tannerella sp. OT286 in the 3 sample clusters analyzed, two from healthy sites and 1 from diseased sites. (DOC) [file pone.0028438.s005.doc]

| **Health Cluster 1** | **Health Cluster 2** | **Disease Cluster 1** | **Disease Cluster 2** |
| --- | --- | --- | --- |
| Desulfobulbus sp. OT041 | Prevotella oris OT311 | Fusobacterium periodonticum OT201 | Actinomyces sp. OT671 688 701 708 |
| Prevotella tannerae OT466 | Streptococcus intermedius and constellatus OT576 644 |  | Actinomyces gerensceriae OT618 |
| Capnocytophaga granulosa and sp. OT325 326 | Prevotella sp. OT658 693 714 782 |  | Bacteroidetes sp. OT274 |
| Actinomyces odontolyticus OT701 | Prevotella nigrescens OT693 |  | Fusobacterium periodonticum OT201 |
| Kingella denitrificans OT582 | Parvimonas micros OT111 |  | Leptotrichia sp. OT462 463 498 563 |
| Prevotella sp. OT317 472 658 | Peptostreptococcus stomatis OT112 |  | Prevotella sp. and II OT317 472 658 |
| Tannerella forsythensis OT613 | Campylobacter gracilis OT623 |  | Prevotella nigrescens OT693 |
| Eubacterium saburreum OT494 | Dialister invisus OT118 |  | Prevotella tannerae OT466 |
| Leptotrichia sp. OT462 463 498 563 |  |  |  |
| TM7 G-1 sp. OT349 346 |  |  |  |
| Rothia dentocariosa and mucilaginosa OT587 681 |  |  |  |
| Prevotella denticola OT291 |  |  |  |
| Selenomonas artemedis OT124 |  |  |  |
| SR1"G-1" sp. OT345 |  |  |  |
| Dialister pneumosintes OT736 |  |  |  |
| Acidaminococcaceae"G-1" sp. OT155 |  |  |  |
| Fusobacterium nucleatum OT202 |  |  |  |
| Gemella morbillorum OT046 |  |  |  |
| Catonella morbi and sp. OT164 165 |  |  |  |
| Rothia dentocariosa OT587 |  |  |  |
| Cardiobacterium valvulum OT540 |  |  |  |
| Prevotella sp. OT658 693 714 782 |  |  |  |
| Eubacterium sp. OT846 |  |  |  |
| Treponema maltophilum OT664 |  |  |  |
| Campylobacter sp. OT580 748 763 |  |  |  |
| Eubacterium yurii OT377 |  |  |  |
| Streptococcus cristatus and sp. OT058 578 |  |  |  |
| Prevotella nigrescens OT693 |  |  |  |
| Selenomonas noxia OT130 |  |  |  |
| Campylobacter showae OT763 |  |  |  |
| Prevotella sp. OT308 |  |  |  |
| Bacteroidetes sp. OT274 |  |  |  |
| Neisseria flavescens OT610 |  |  |  |
| Megasphaera micronucliformus OT122 |  |  |  |
| Prevotella oulora OT288 |  |  |  |
| Selenomonas sp. OT126 479 481 639 |  |  |  |
| Selenomonas sp. OT138 146 |  |  |  |
| Selenomonas infelix OT639 |  |  |  |
